# Response of leaf photosynthesis, chlorophyll content and yield of hydroponic tomatoes to different water salinity levels

**DOI:** 10.1371/journal.pone.0293098

**Published:** 2024-02-09

**Authors:** Khalid A. Al-Gaadi, ElKamil Tola, Rangaswamy Madugundu, Ahmed M. Zeyada, Ahmed A. Alameen, Mohamed K. Edrris, Haroon F. Edrees, Omer Mahjoop

**Affiliations:** 1 Department of Agricultural Engineering, College of Food and Agriculture Sciences, King Saud University, Riyadh, Saudi Arabia; 2 Precision Agriculture Research Chair, Deanship of Scientific Research, King Saud University, Riyadh, Saudi Arabia; Bahauddin Zakariya University, PAKISTAN

## Abstract

Tomato (*Solanum lycopersicum* L.) is an important vegetable crop that grows easily under controlled conditions, such as in greenhouses and hydroponics. To overcome freshwater scarcity, researchers are searching for alternatives to groundwater sources such as desalinated water (saline water) for irrigation. High salinity in irrigation water alters physiological functions and crop development, thereby reducing the yield. Best management practices and the use of grafted tomato plants on salt-tolerant rootstocks can alleviate salinity stress. The present study was conducted to address the impact of salinity stress on leaf transpiration (Tr), stomatal conductance (gs), photosynthesis (Pn), leaf chlorophyll content, proline content, and yield of hydroponically cultivated tomato plants. Saline (NaCl) water was used for the preparation of nutrient solution with three salinity levels, electrical conductivity (EC, dS m^−1^) of 2.5 (control), 6.0, and 9.5. Three commercial tomato cultivars (Valouro-RZ, Ghandora-F1, and Feisty-Red) were used. Both self-rooted plants and plants grafted onto Maxifort rootstocks were transplanted onto a perlite substrate. The recorded data revealed that all studied cultivars were critically affected by higher salinity (≈ 9.5 dS m^-1^) compared to low (≈ 2.5 dS m^-1^) and medium (≈ 6.0 dS m^-1^) salinity levels. The Variations in Tr, Pn, gs, chlorophyll content of leaf, and yield between medium and high salinity trials were reported at 3%, 5%, 9%, 5%, and 7.1%, respectively, whereas no significant differences were observed between low and medium salinities. However, at medium salinity levels, grafted plants performed better in photosynthesis than non-grafted plants. This is due to the accumulation of leaf proline, which maintains osmotic regulation and photosynthetic activity by preventing cell damage at medium salinities. Hence, this study confirmed the use of saline water for growing tomatoes under hydroponic conditions up to an EC of 6.0 dS m^-1^ including the EC of nutrient fertilizers.

## Introduction

Salinity is a major abiotic stress that has gained global interest for its negative effects on crop growth and reduced productivity [1]. Salt stress critically affects the plant growth rate by altering a variety of physiological functions depending on several crop factors (i.e., plant variety, growth stage, and salt concentration) [2]. Furthermore, salinity stress generates osmotic shock in the root zone, alters the water-uptake process, and causes an imbalance in nutrient uptake and translocation, which lead to a decline in crop yield [3]. Salinity also hinders photosynthetic activity, weakens stomatal conduction and carbon dioxide supply, and affects the soil-plant-atmosphere continuum, thus altering photosynthetic carbon assimilation and related physiological processes [4]. Moreover, studies have reported the occurrence of diseases and pests owing to high salinity stress and stunted growth [5, 6].

Nasrin and Mannan [7] reported that the administration of saline practices for growing crops in fields or greenhouses would be costly and temporary; therefore, the initiatives on salt tolerance practices are a good method to limit the adverse consequences of salinity stress on plants and enhance production. Therefore, studies on the use of saline water for irrigation could be a profitable agricultural practice for sustainable greenhouse production and self-sufficiency in food production [8].

Protected agriculture is a technology-driven agricultural practice that optimizes the climatic conditions suitable for a crop throughout the growing season for food security. This can be achieved by establishing greenhouses using modern techniques such as hydroponics [9]. Hydroponics is a soilless cultivation method that uses nutrient solutions (recipes) for fertigation and plant-anchoring substrates such as perlite, rock wool, clay pellets, peat moss, and vermiculite, instead of soil [10]. It is widely used to improve yield through better management of agricultural inputs in controlled environments [10].

Worldwide commercial production of tomato (*Solanum lycopersicum* L.) has become the second most important vegetable crop after potato [11], with global production estimated at 186,821 million kilos [12]. Tomatoes are the largest hydroponically produced crop worldwide and one of the major crops cultivated in greenhouses in the Kingdom of Saudi Arabia (KSA), with 1260 hectares of greenhouse planted area and average productivity of 8–10 kg m^-2^ and 35–45 kg m^-2^ under uncontrolled and controlled greenhouses, respectively [13]. The production of tomatoes in the KSA in 2021 is estimated at 28.5 t ha^-1^ and 112 t ha^-1^ under conventional and protected agriculture practices, respectively [13]. Tomato crops are moderately sensitive to salinity stress. The magnitude of tolerance to salinity stress depends mainly on the biophysics and genetics of the cultivars and the management practices [14]. As per the study conducted by Zhang et al. [15], photosynthesis is a vital physiological process, which explains the growth and behavior of the tomato plant under salinity stress.

Another study [16] also highlighted the importance of leaf photosynthesis measurements for monitoring crops cultivated under saline conditions, and there is evidence that salt stress influences key photosynthetic enzyme activities as well as chlorophyll and carotenoid contents [17]. Photosynthesis is sensitive to salt stress because of the rapid stomatal closure; however, the energy produced by photosynthesis is transformed from growth to stress defense [4]. Salt-induced reduction in leaf photosynthesis is due to stomatal closure, decreased concentrations of intracellular CO_2_ and other non-stomatal characteristics [18]. The stomatal closure restricts the phenomenon of transpiration and gas exchange, limits the availability of intercellular CO_2_ (Ci), and alters leaf biochemistry; collectively affecting the net photosynthetic rate (Pn) under prolonged salt stress conditions. Moreover, the stomatal conductance (gs) and Pn decreased concurrently under various saline conditions [4]. However, high salinity may cause changes in leaf autonomy resulting from stomatal closure to minimize the transpiration rate, thus affecting growth development, photosynthetic efficiency, and physiological damage [19]. Salinity stress also alters the plant-water relationships and nutrient uptake of tomato plants, resulting in the malfunction of cell development and vital physiological activities, such as chlorophyll production, stomatal rhythms, and photosynthesis. To overcome abiotic stress, plants induce the accumulation of proline [20] and soluble sugars [21] to safeguard cell membranes by managing intercellular osmotic pressure. Moreover, biomass production in plants is correlated with energy consumption and gas exchange, which in turn depend on net photosynthesis [22]. In view of the food security and sustainability of available water resources, Saudi Arabia is focusing on alternative irrigation resources for groundwater sources, such as desalinated water (i.e., saline water). Hence, the main goal of this study was to assess the effect of salinity stress on the rate of photosynthesis (Pn), chlorophyll content, and yield of tomato plants grown under hydroponics, with the use of saline water for irrigation. This study hypothesized that induced salinity treatments destroy chlorophyll and alter photosynthesis, resulting in stunted plant growth and reduced production. In response to salinity stress, tomato plants trigger proline accumulation to manage their cell structure and physiological properties [20, 23]. Hence, the quantification of leaf proline, photosynthetic parameters (Tr, Pn, and gs), and chlorophyll content will provide information on the impact of salinity stress on tomato plants irrigated with saline water.

## Materials and methods

### Experimental setup

**Fig 1:**
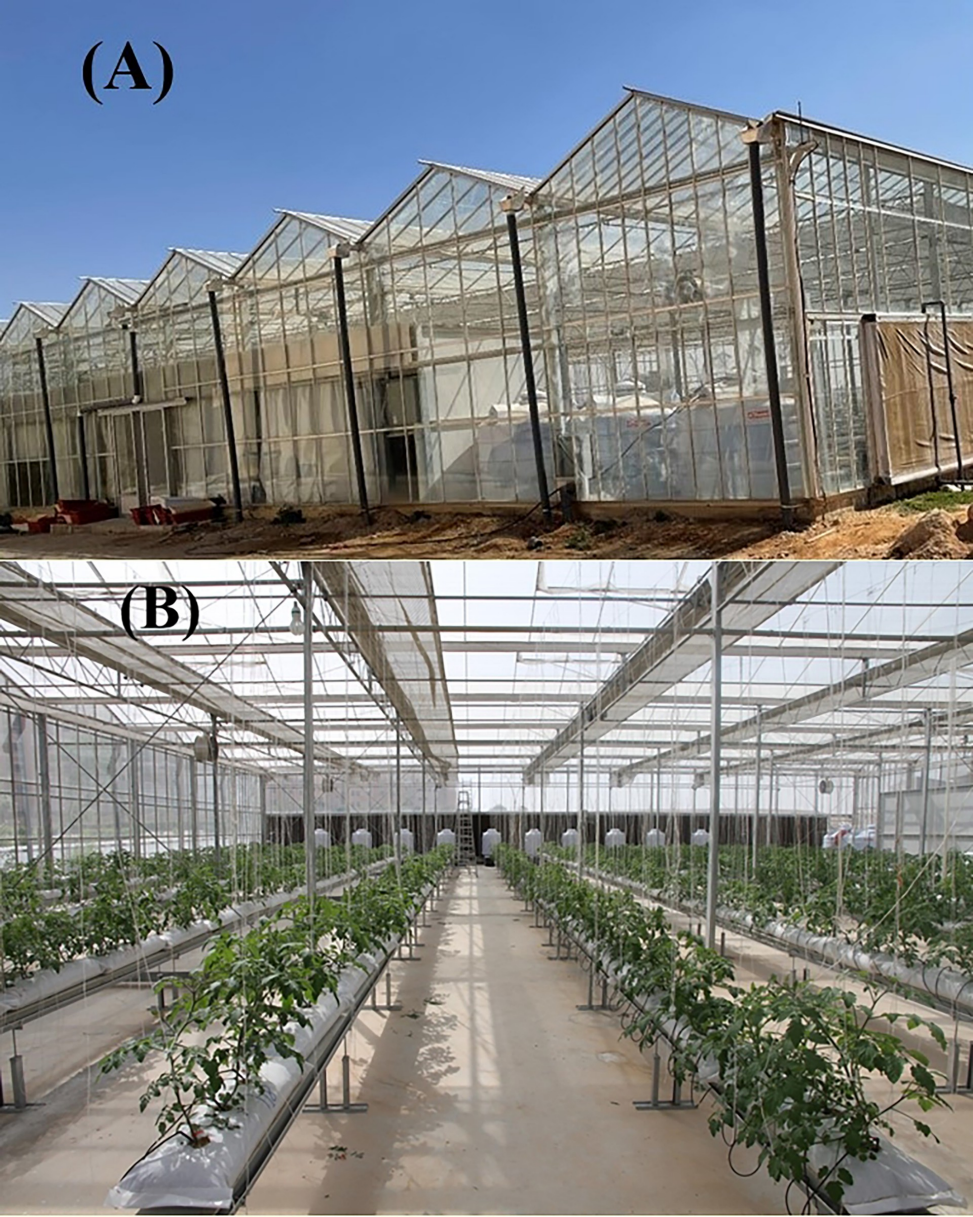
(A) Hydroponic greenhouse structure, (B) Plant lines (steel troughs) with perlite bags and irrigation system.

Three tomato cultivars, Valouro-RZ, Ghandora-F1, and Feisty-Red, were grown on perlite substrates. A hydroponic system installed in a greenhouse was utilized for the cultivation of tomato plants and the implementation of experimental trials. The greenhouse (Fig 1) is located in the Educational farm, College of Food and Agriculture Sciences, King Saud University, Riyadh, Saudi Arabia (46° 37′ 10″ N & 24° 44′ 12″ E). Tomato seedlings (both self-rooted/non-grafted and grafted) were prepared at the ASDCO Nurseries, Al-Kharj, Saudi Arabia. Scions of selected cultivars were grafted over a rootstock, Maxifort (*S*. *lycopersicum × S*. *habrochaites*), using a tube grafting procedure [24]. A MACQU hydroponic (Geosmart, Athens, Greece) system was installed to optimize the indoor climate, fertilizer dosing, and irrigation practices. The greenhouse (28 m x 32 m x 4.5 m) has been supplied with 12 plant lines of stainless-steel troughs of 1.0 m in height and a slope of 5%, and ≈1.78 m between every two lines. Perlite bags (0.90 m × 0.22 m × 0.15 m) were used as substrate and self-draining drippers, with a discharge capacity of 3 L h^-1^ were administered to supply nutrient solution through the “MACQU.STP” software program.

The prepared seedlings were transplanted onto a perlite substrate on November 24, 2021, at a density of 2.25 plants m^-2^ and 25 cm between the two plants was maintained. The nutrient solution was supplied to the experimental plants at three different salinity levels (EC, dS m^−1^), 2.5 (salinity-1, control), 6.0 (salinity-2), and 9.5 (salinity-3). For the preparation of control nutrient solution, i.e. 2.5 dS m^−1^ (salinity-1), water-soluble fertilizers, which contained in mmol l^−1^: NO_3_^−^ 13.5; NH_4_^+^ 1.5; PO_4_^3−^ 1.25; K^+^ 8.75; Ca_2_^+^ 4.25; Mg^2+^ 2.0; SO_4_^2−^ 3.75, were dissolved in a municipality supplied water with the EC of 0.5. i.e., the EC of 2.0 dS m^-1^ from the fertilizers and EC of municipal water (0.5 dS m^-1^) together reached the required EC of 2.5 dS m^-1^ for the salinity-1 (control) treatments. In the case of induced salinity treatments 6.0 dS m^−1^ (salinity-2) and 9.5 dS m^−1^ (salinity-3), the EC of 2.0 dS m^-1^ was maintained from the fertilizers, and the rest of the EC was achieved by adding the 40mM and 70mM of NaCl, respectively, as a stress inducer. To manage possible osmotic shock, the NaCl concentration was initially 20 mM and gradually increased to the target concentration. The pH of the nutrient solution was maintained at 5.5–6.5 throughout the study. This study will be conducted until the end of March 2022. The experimental greenhouse was located in an arid climate with high temperatures and long day lengths (10:40 h). in winter to 13:40 hrs. in summer) The hydroponic greenhouse was controlled (temperature, relative humidity, and light sensors). Throughout the study, the greenhouse temperature was maintained between 18°C and 22°C during daytime. Solar radiation was maintained at a flux of 400 nm. Automated shading screens were used to manage the light dispersion during solar hours. A fan pad cooling system and a heating system were used to maintain the temperature inside the greenhouse. The nutrient solution was delivered to the plants for 2 min (initial growth stage) to 8 min (maturity stage) every 2–7 d during the daytime using automatic activated motor pumps. During the night, water was supplied twice (3 min each) during high-temperature days (i.e., > 34°C outside the greenhouse), one at 11 p.m. and the other at 2 a.m.

### Leaf photosynthesis parameters

Leaf photosynthesis parameters, such as transpiration rate (Tr), net photosynthetic rate (Pn), and stomatal conductance (gs), were measured between 8–11 a.m. to avoid environmental midday stresses. Measurements were taken at a flow rate of 500 μmol s^-1^ from three fully opened young leaves in each plant, with an average of 20 readings per measurement, using a portable photosynthesis system (LI-6400XT, Li-Cor Inc., Lincoln, NE, USA).

### Leaf chlorophyll content

The chlorophyll content or greenness of tomato leaves, which is used as an indicator of tomato health, was recorded along with photosynthetic measurements from three plants as an average of three leaves from each plant. Measurements were taken twice, 60 days and 120 days after transplantation, using a SPAD meter (model, 502 Plus, Spectrum Technologies, Aurora, Illinois, USA).

### Proline concentration

According to Claussen [20], proline accumulation data can provide information on the physiological status of plants under abiotic or salinity stress. The leaf proline content was determined using the colorimetric assay described by Abraham et al. [25]. Fresh tomato leaves were used for the assessment, and the absorbance of the supernatant of the leaf extract was measured at 520 nm using toluene as a reference and expressed as micrograms per gram of fresh weight.

### Tomato fruit yield

Every seven days, the tomato fruits that have reached at least 80% red ripeness stage were harvested, and then weighed using a balance scale (± 0.05 accuracy). The tomatoes were harvested at different times, and the total yield (kg m^-2^) of the plants was recorded and segregated according to the cultivar, grafting conditions, and induced salinity levels for statistical analysis.

### Statistical analysis

A split-split plot design analysis was performed with three different salinity concentrations as the main treatments, grafting (non-grafted and grafted) as the sub-treatments, and two measurement days after transplanting (DAT) as a sub-sub treatment, with three replicates. The ANOVA procedure within the Statistical Analysis System (SAS for Windows v. 9.4) was used to determine the interaction effects of the different salinity concentrations on leaf photosynthesis parameters, chlorophyll content of leaves, and tomato fruit yield.

## Results and discussion

### Leaf photosynthesis

Leaf photosynthesis parameters, such as transpiration rate (Tr), net photosynthetic rate (Pn), and stomatal conductance (gs), were critically affected by salinity levels (S1 Data). The responses of the leaf photosynthetic parameters of tomato cultivars to salinity, grafting, and crop age are discussed in the following sections.

#### Transpiration rate (Tr)

**Fig 2:**
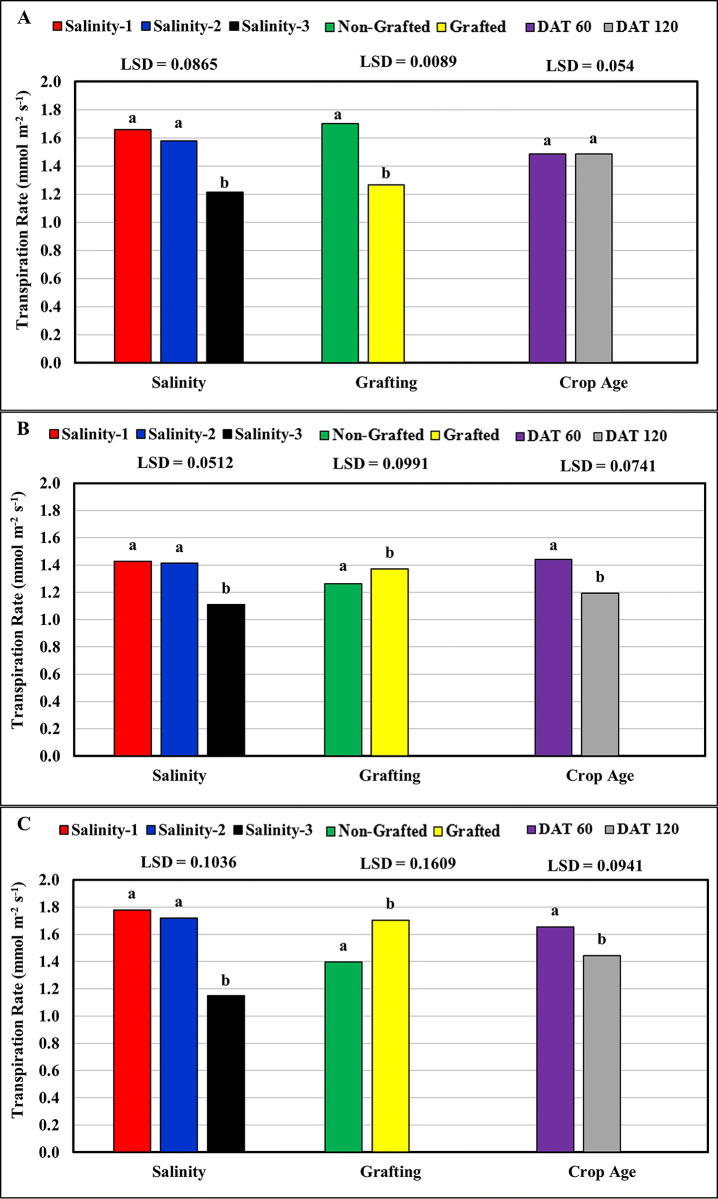
Response of the leaf transpiration rate to salinity, grafting and crop age: (A) Valouro-RZ, (B) Ghandora-F1, and (C) Feisty-Red.

The results shown in Table 1 and Fig 2 show an inverse and statistically significant correlation (Pr<0.0001) between salinity and leaf transpiration rate (Tr). This is attributed to the fact that when plants are under salt stress the Tr is limited and the tension that pushes water through the apoplast pathway is reduced [26]. Where the mean Tr value (1.16 mmol m^-2^ s^-1^) at high salinity level (salinity-3 ≈ 9.5 dS m^-1^) was found significantly lower than that at both low (salinity-1 ≈ 2.5 dS m^-1^) and medium (salinity-2 ≈ 6.0 dS m^-1^) salinity levels, with mean Tr values of 1.62 mmol m^-2^ s^-1^ and 1.57 mmol m^-2^ s^-1^, respectively. No significant differences were found between the Tr values at low and medium salinities. The Tr results obtained for tomato plants were consistent with those reported by Tang et al. [27], where the leaf transpiration rate decreased with increasing salinity levels. On average, Feisty-Red showed the highest mean Tr value (1.55 mmol m^-2^ s^-1^) among the studied tomato cultivars, followed by Valouro-RZ (1.48 mmol m^-2^ s^-1^), and the low values were recorded for Ghandora-F1 (1.32 mmol m^-2^ s^-1^). Grafting the tomato cultivars on the Maxifort rootstock resulted in a significant improvement in the mean Tr values for both the Ghandora-F1 and Feisty-Red cultivars. For the Valouro-RZ cultivar, grafted plants had significantly lower mean Tr values than non-grafted plants (Fig 2). However, a decrease was recorded in the mean Tr values at 120 days after transplanting (DAT) compared with those recorded at 60 DAT). Where the mean Tr values at 120 DAT for both Ghandora-F1 (1.19 mmol m^-2^ s^-1^) and Feisty-Red (1.45 mmol m^-2^ s^-1^) cultivars were significantly lower compared to those recorded at 60 DAT with mean values of 1.44 and 1.65 mmol m^-2^ s^-1^, respectively. However, the mean Tr values were found to be stable for the Valouro-RZ cultivar, with no significant differences between the measurements recorded at 60 DAT (1.48 mmol m^-2^ s^-1^) and those at 120 DAT (1.49 mmol m^-2^ s^-1^).

**Table 1:** Statistical results: Effect of salinity, grafting, and crop age on the leaf transpiration rate.

	Source	Valouro-RZ	Ghandora-F1	Feisty-Red
**Pr > F**	Gra	<0.0001	<0.0001	<0.0001
	DAT	0.972	<0.0001	<0.0001
	Gra × DAT	0.0014	0.0012	0.0092
	Sal	<0.0001	<0.0001	<0.0001
	Gra × Sal	<0.0001	0.0276	0.0009
	DAT × Sal	0.1155	0.0001	<0.0001
	Gra × DAT × Sal	<0.0001	0.0068	<0.0001
**R-Square**		0.96	0.97	0.97
**CV**		6.73	4.49	7.73
**RMSE**		0.099	0.059	0.119

Gra = Grafting, DAT = Days After Transplanting, Sal = salinity, CV = Coefficient of Variation, RMSE = Root Mean Square Error.

#### Net photosynthetic rate (Pn)

**Fig 3:**
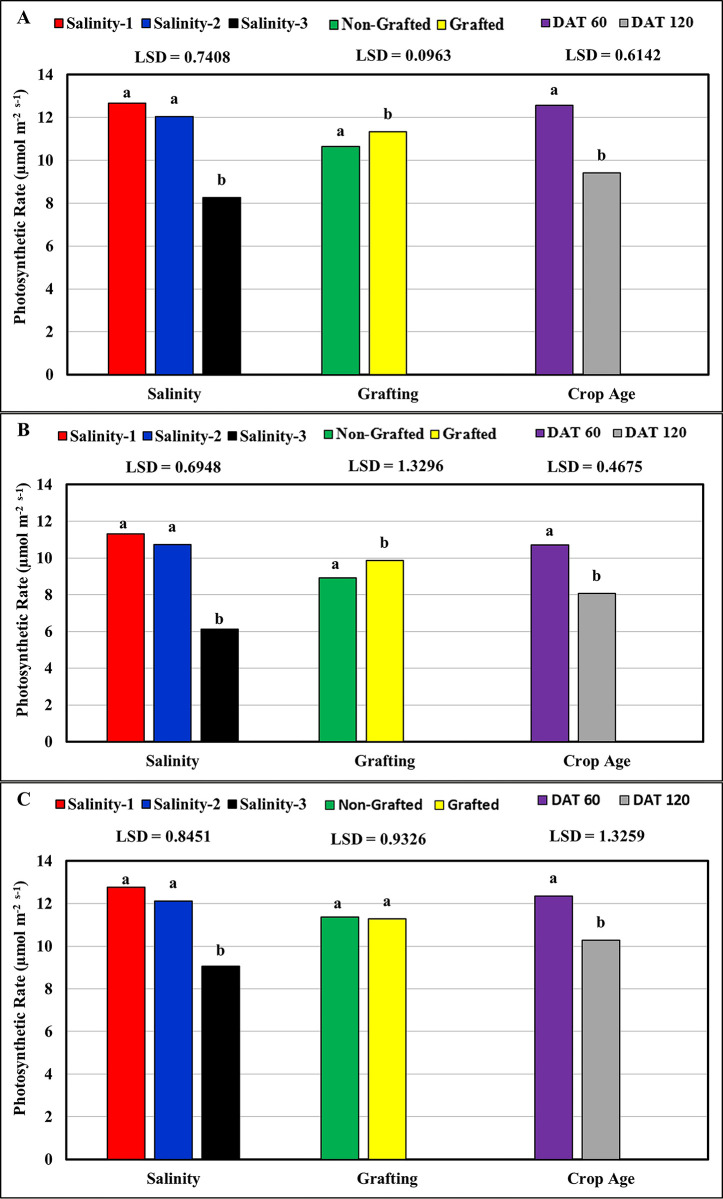
Response of the photosynthetic rate to salinity, grafting and crop age: (A) Valouro-RZ, (B) Ghandora-F1, and (C) Feisty-Red.

The results presented in Table 2 and Fig 3 indicate that the net photosynthetic rate (Pn) of the tomato cultivars studied responded significantly to salinity, grafting, and crop age, except for the Feisty-Red cultivar, in which Pn showed no significant response to grafting. The results showed an inverse correlation with salinity concentration, as the highest (12.25 μmol m^-2^ s^-1^) and the lowest (7.81 μmol m^-2^ s^-1^) mean Pn values were recorded at the low salinity (salinity-1 ≈ 2.5 dS m^-1^) and the high salinity (salinity-3 ≈ 9.5 dS m^-1^), respectively. However, the statistical results showed significant differences in Pn values only between the high salinity (salinity-3) and both the low salinity (salinity-1) and the medium salinity (salinity-2 ≈ 6.0 dS m^-1^), but no significant differences were observed between the Pn values at the low and medium salinities for the three tomato cultivars. In general, the cultivar Feisty-Red showed the best salinity tolerance based on the highest average Pn value (11.32 μmol m^-2^ s^-1^), followed by Valouro-RZ (10.98 μmol m^-2^ s^-1^), while Ghandora-F1 showed the least values (9.39 μmol m^-2^ s^-1^). On other hand, the mean photosynthetic rate of grafted plants was 10.85 μmol m^-2^ s^-1^, which was significantly more than the mean value of non-grafted pants (10.28 μmol m^-2^ s^-1^) for Valouro-RZ and Ghandora-F1 cultivars, while for Feisty-Red cultivar, the mean value of non-grafted plants (11.36 μmol m^-2^ s^-1^) was slightly higher than grafted plants (11.27 μmol m^-2^ s^-1^), but no significant difference between them. The temporal difference in transpiration between DAT 60 and DAT 120 indicated that the values of the Pn rate were better at DAT 60 than at DAT 120, as shown in Fig 3. This is because plants that are under salt stress for a long period of time experience ionic toxicity because of the imbalance in cytoplasmic nutrition, which in turn leads to a decrease in the photosynthesis rate [28].

**Table 2:** Statistical results: Effect of salinity, grafting, and crop age on the photosynthetic rate.

	Source	Valouro-RZ	Ghandora-F1	Feisty-Red
**Pr > F**	Gra	0.0298	0.0025	0.795
	DAT	<0.0001	<0.0001	<0.0001
	Gra*DAT	0.1074	0.9015	0.0046
	Sal	<0.0001	<0.0001	<0.0001
	Gra*Sal	0.0940	0.3992	0.0056
	Dat*Sal	<0.0001	0.1398	<0.0001
	Gra*Dat*Sal	0.1525	0.1786	0.0555
**R-Square**		0.96	0.96	0.95
**CV**		7.79	8.55	8.62
**RMSE**		0.855	0.802	0.976

Gra = Grafting, DAT = Days After Transplanting, Sal = salinity, CV = Coefficient of Variation, RMSE = Root Mean Square Error.

Grafting tomato plants onto Maxifort significantly improved the Pn for both Valouro-RZ (Pr<0.0298) and Ghandora-F1 (Pr<0.0025) cultivars. In the Feisty-Red cultivar, grafting showed no significant improvement in Pn. Moreover, crop age exhibited a significant negative effect on Pn (Pr<0.0001) in all three tomato cultivars. Tuzet [29] reported that transpiration and photosynthesis rates were positively correlated. However, any negative effect on photosynthesis in leaves critically affects water-use efficiency in the transpiration process.

#### Stomatal conductance (gs)

**Fig 4:**
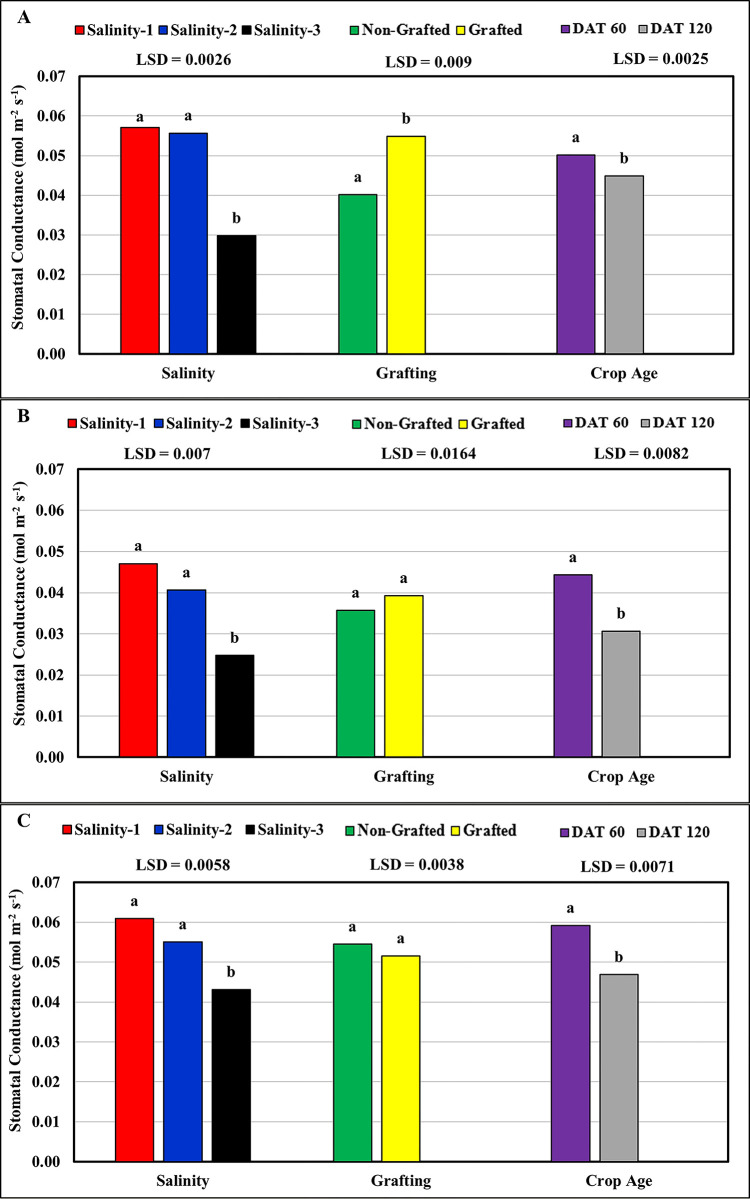
Response of stomatal conductance to salinity, grafting and crop age: (A) Valouro-RZ, (B) Ghandora-F1, and (C) Feisty-Red.

These results indicated that an increase in salinity led to a decrease in stomatal conductance. However, the statistical results (Table 3) showed that values of the stomatal conductance at high salinity (salinity-3 ≈ 9.5 dS m^-1^) were significantly lower (Pr<0.0001) compared to that at the low (salinity-1 ≈ 2.5 dS m^-1^) and medium (salinity-2 ≈ 6.0 dS m^-1^) salinities for all tomato cultivars (Fig 4). However, no statistically significant differences in stomatal conductance were observed between the low- and medium-salinity treatments. The highest average stomatal conductivity (0.052 mol m^-2^s^-1^) was recorded for the Feisty-Red cultivar, followed by Valouro-RZ (0.047 mol m^-2^s^-1^), the lowest values were recorded for the Ghandora-F1 cultivar (0.037 mol m^-2^s^-1^). Moreover, grafting onto Maxifort improved the stomatal conductance of the tomato cultivars studied, but significant differences were recorded between the non-grafted and grafted plants only for the Valouro-RZ cultivar. The stomatal conductance results showed an inverse correlation with crop age, where the results recorded at 60 DAT were significantly higher (Pr<0.0001) than those recorded at 120 DAT for all tomato cultivars studied. This is because an increase in salt concentration leads to stomatal closure, which in turn reduces photosynthesis [30]; thus, the rate of photosynthesis and stomatal conductance decrease in conjunction with an increase in salinity concentration [31]. Chaves et al. [4] reported that stomatal closure due to salinity stress generally occurs as a result of decreased leaf turgor and atmospheric vapor pressure, along with chemical signals generated by the roots.

**Table 3:** Statistical results: Effect of salinity, grafting, and crop age on the stomatal conductance.

	Source	Valouro-RZ	Ghandora-F1	Feisty-Red
**Pr > F**	Gra	<0.0001	0.231	0.226
	DAT	<0.0001	0.0001	<0.0001
	Gra*DAT	0.0334	0.4421	0.0178
	Sal	<0.0001	<0.0001	<0.0001
	Gra*Sal	<0.0001	0.8321	0.6482
	Dat*Sal	<0.0001	0.3118	0.0093
	Gra*Dat*Sal	0.0010	0.9009	0.2468
**R-Square**		0.985	0.848	0.866
**CV**		6.409	21.457	12.753
**RMSE**		0.003	0.008	0.006

Gra = Grafting, DAT = Days After Transplanting, Sal = salinity, CV = Coefficient of Variation, RMSE = Root Mean Square Error.

### Chlorophyll content

**Fig 5:**
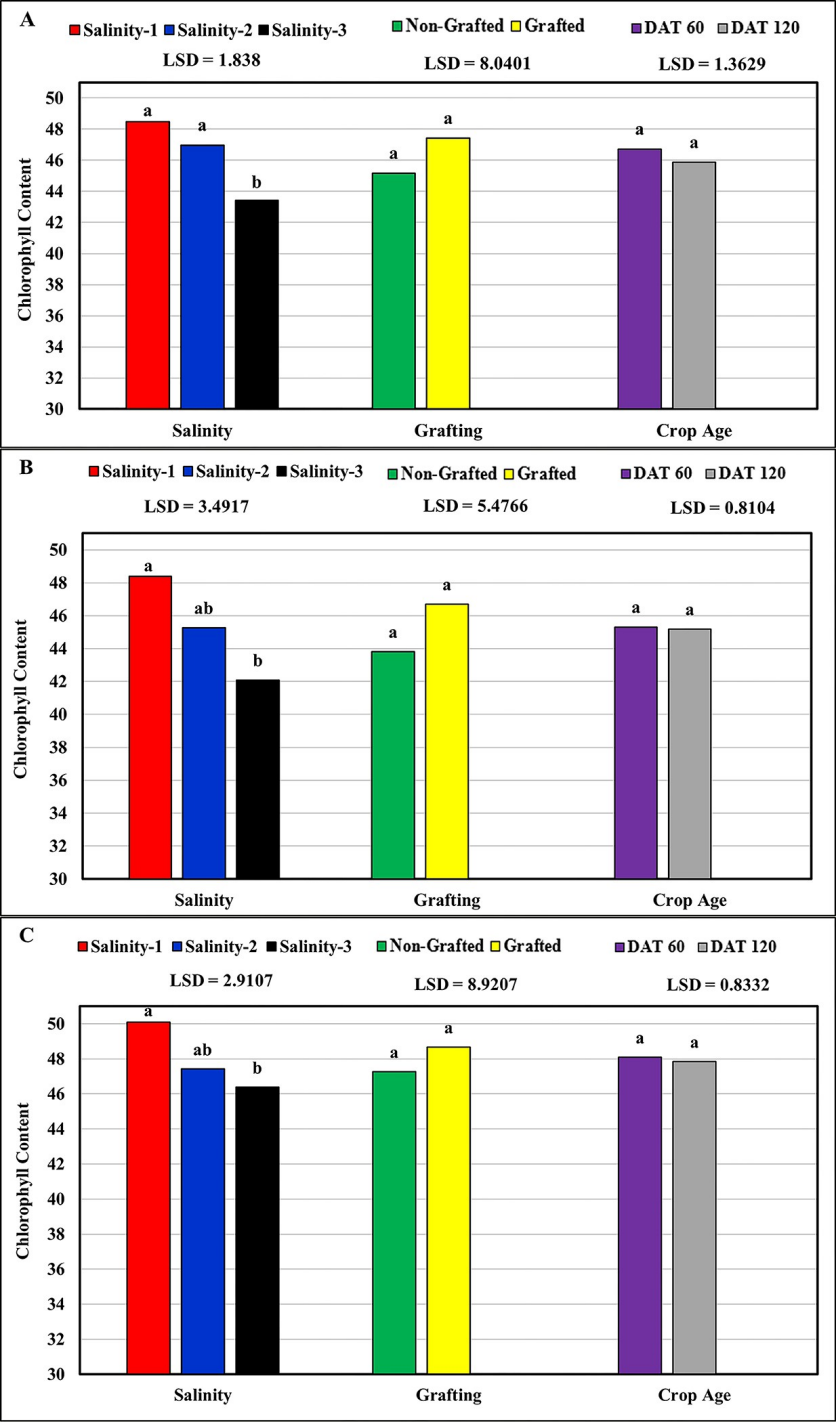
Response of chlorophyll content to salinity, grafting and crop age: (A) Valouro-RZ, (B) Ghandora-F1, and (C) Feisty-Red.

The results presented in Table 4 showed that the chlorophyll content of the leaves was critically affected by salinity (Pr<0.0001) for all the studied tomato cultivars. Where, the highest (48.99) and the lowest (43.95) mean values were recorded at low salinity (salinity-1 ≈ 2.5 dS m^-1^) and high salinity (salinity-3 ≈ 9.5 dS m^-1^), respectively. The statistical analysis results showed that significant differences in the chlorophyll content were recorded only between the high salinity (salinity-3 ≈ 9.5 dS m^-1^) and both the low salinity (salinity-1 ≈ 2.5 dS m^-1^) for the three tomato cultivars, and the medium (salinity-2 ≈ 6.0 dS m^-1^) salinity only for the cultivar Valouro-RZ (Fig 5). These results agree with the findings of Parvin et al. [32], who found a significant decrease in the total chlorophyll content at a salinity level of 8 dS m^–1^ and a slight increase at salinity levels ranging between 2 and 4 dS m^–1^. The chlorophyll content was significantly reduced at 6 dS m^–1^, these results were also in agreement with the results of [15, 33, 34], who stated that salinity reduced the total chlorophyll content in tomato leaves.

**Table 4:** Statistical results: Effect of salinity, grafting, and crop age on the chlorophyll content of the leaf.

	Source	Valouro-RZ	Ghandora-F1	Feisty-Red
**Pr > F**	Gra	0.005	0.0474	0.226
	DAT	0.242	0.9204	0.826
	Gra*DAT	0.6828	0.7848	0.7316
	Sal	< .0001	0.0054	0.041
	Gra*Sal	0.0515	0.9982	0.4478
	Dat*Sal	0.3869	0.8560	0.8558
	Gra*Dat*Sal	0.9892	0.9917	0.9596
**R-Square**		0.83	0.58	0.55
**CV**		4.59	8.92	7.03
**RMSE**		2.12	4.03	3.36

Gra = Grafting, DAT = Days After Transplanting, Sal = salinity, CV = Coefficient of Variation, RMSE = Root Mean Square Error.

In general, the cultivar Feisty-Red had the highest mean chlorophyll content value (47.96), followed by Valouro-RZ (46.28), while the lowest values were recorded for Ghandora-F1 (45.24). Moreover, the overall mean chlorophyll content of grafted plants (46.84) was higher than that of non-grafted plants (46.16) for all varieties (Fig 5). However, the chlorophyll content values at DAT 60 were higher than those measured at DAT 120, with no significant differences between them. This may be attributed to the fact that the accumulation of Na + and Cl-ions increased after transplanting, leading to an increase in salt concentration, which in turn led to increased salinity levels; however, the speed of germination and germination energy decreased and had negative effects on chlorophyll and total yield [35].

### Tomato fruit yield

**Fig 6:**
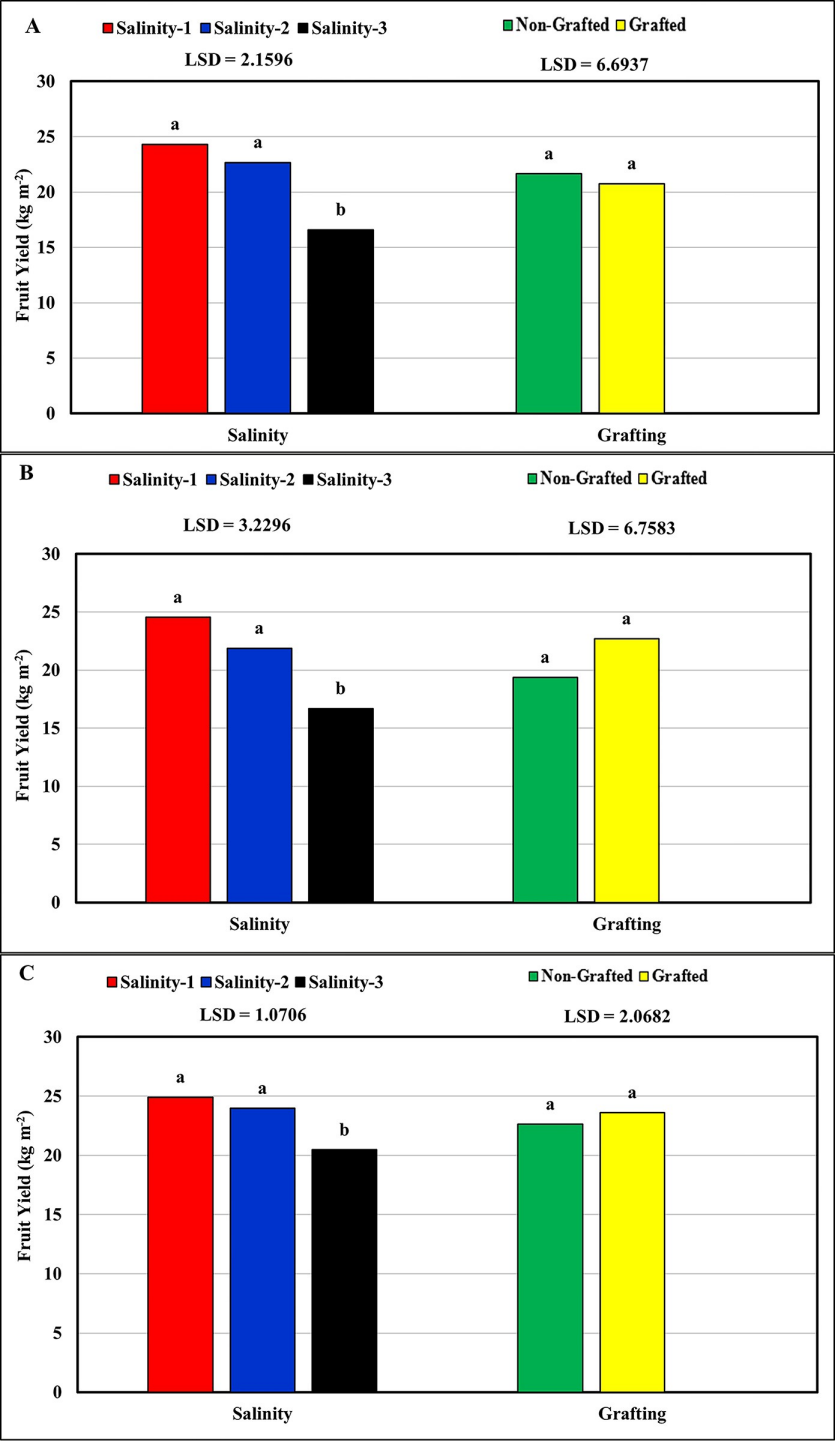
Response of tomato fruit yield of the three studied tomato cultivars to salinity and grafting: (A) Valouro-RZ, (B) Ghandora-F1, and (C) Feisty-Red.

The results showed a negative impact of salinity on the total tomato fruit yield, with the highest (24.59 kg m^-2^) and lowest (17.92 kg m^-2^) mean fruit yield recorded at low salinity (salinity-1 ≈ 2.5 dS m^-1^) and high salinity (salinity-3 ≈ 9.5 dS m^-1^), respectively. However, the statistical results (Table 5) showed that significant differences in the tomato fruit yield were recorded only at high salinity (salinity-3 ≈ 9.5 dS m^-1^) compared with low (salinity-1 ≈ 2.5 dS m^-1^) and medium (salinity-2 ≈ 6.0 dS m^-1^) salinities for all tomato cultivars (Fig 6). No significant differences in tomato fruit yield were observed between low and medium salinities. However, salinity affects fruit size, which is reflected in the fresh fruit yield under high salinity. Although grafting tomato plants onto the Maxifort rootstock improved the total fruit yield of the studied cultivars, no significant differences in the total fruit yield between the non-grafted and grafted plants were recorded. The fruit yields of the grafted plants (22.35 kg m^-2^) were higher than that of the non-grafted plants (21.21 kg m^-2^) for Valouro-RZ, Ghandora-F1, and Feisty-Red.

**Table 5:** Statistical results: Effect of salinity and grafting on the total tomato fruit yield.

	Source	Valouro-RZ	Ghandora-F1	Feisty-Red
**Pr > F**	Gra	0.3014	0.016	0.030
	Sal	< .0001	0.0003	< .0001
	Gra*Sal	0.2058	0.2759	0.5478
**R-Square**		0.85	0.74	0.86
**CV**		11.78	17.74	5.35
**RMSE**		2.49	3.73	1.24

Gra = Grafting, DAT = Days After Transplanting, Sal = salinity, CV = Coefficient of Variation, RMSE = Root Mean Square Error.

These results are consistent with the findings of [27], who reported that with increasing salt concentrations, the transpiration rate, net photosynthesis rate, stomatal conductance, and chlorophyll content decreased, leading to a decrease in the total yield. The negative effect of salt stress on tomatoes is a result of retarded plant growth owing to a decrease in the photosynthetic rate [36], which leads to a reduction in fruit size and total yield per plant. Moreover, many studies have indicated that high salt concentrations in tomato plants, especially in the root zone, negatively affect plant growth and fruit size, leading to reduced fruit yield [5]. In addition, tomato plants under salt stress may experience reduced water potential in the root zone, causing water deficits [2].

### Effect of saline water use on photosynthesis parameters and yield

**Fig 7:**
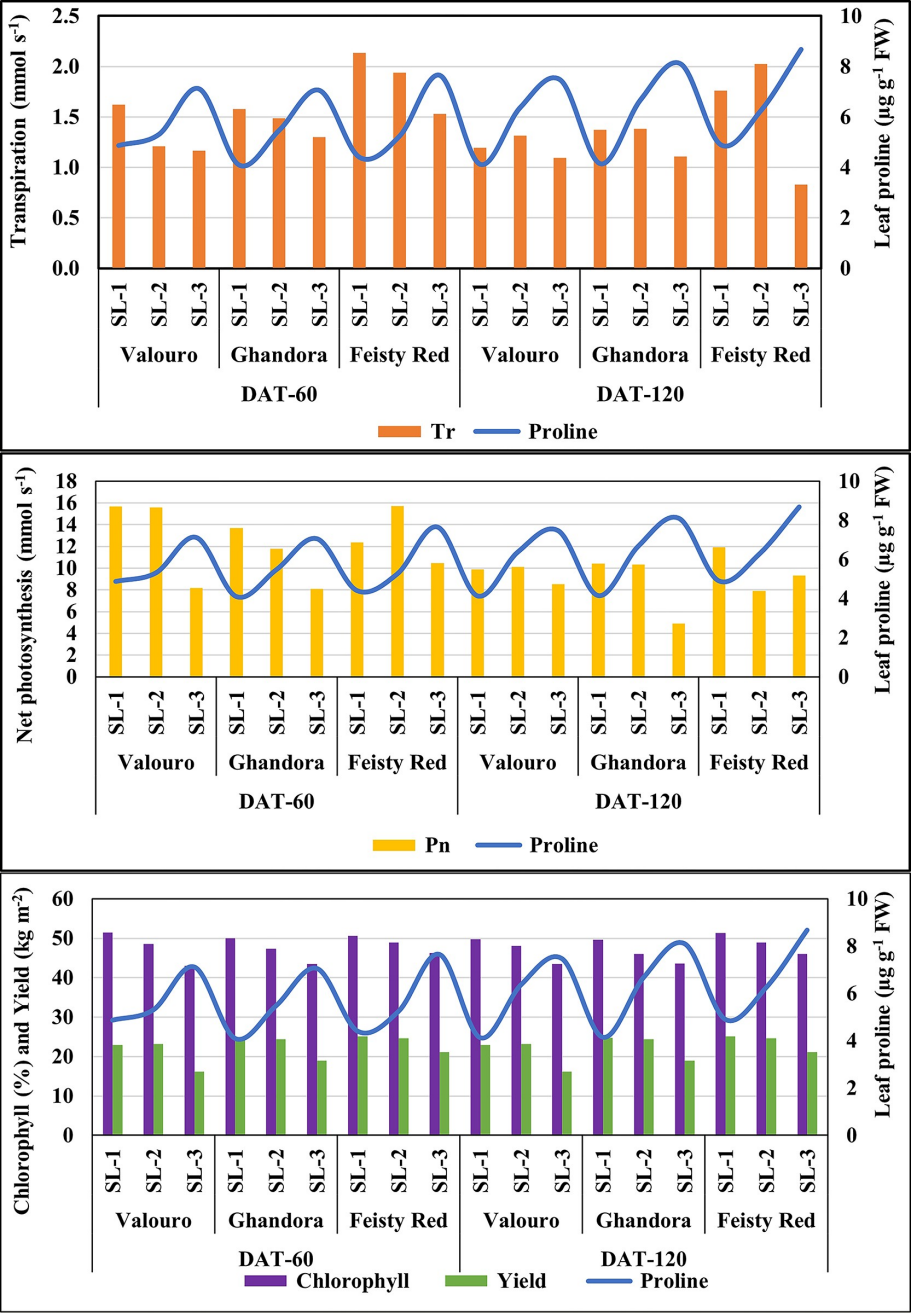
Effect of saline water use on photosynthesis parameters and yield of grafted plants.

**Fig 8:**
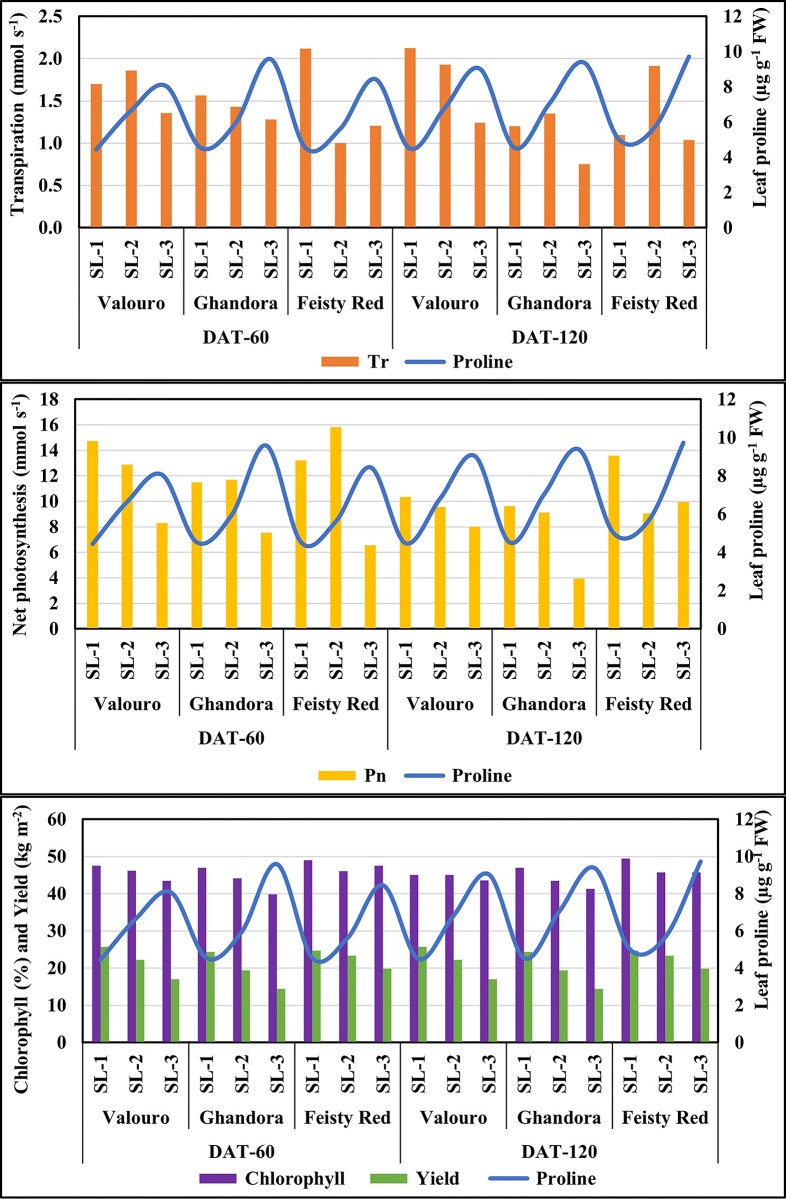
Effect of saline water use on photosynthesis parameters and yield of non- grafted plants.

Different EC levels of saline water used for irrigation significantly affected photosynthetic parameters and showed a negative impact of salinity on Tr, gs, Pn, and chlorophyll content at both DAT 60 and DAT 120 (S2 Data). In response to salinity stress, the accumulation of proline in tomato leaves was observed in both grafted (Fig 7) and non-grafted plants (Fig 8). Leaf proline significantly accumulated with increasing EC in the saline water used for irrigation. The highest proline content was achieved in the leaves of non-grafted plants compared to grafted plants. The maximum accumulation (7.7 μg g^-1^ FW) of proline was observed in grafted plants, while the leaf proline in non-grafted plants was reported at 9 μg g^-1^ FW at higher salinity (9.5 dS m^-1^). A similar trend of proline was noticed with the plants treated with medium (6.0 dS m^-1^) saline water. No significant differences in leaf proline content were observed in plants treated with low-salinity or control irrigation water. These results are in agreement with those of Yanyan et al. [37] and Al-Harbi et al. [38], who reported increasing amounts of leaf proline with increased abiotic stress by salinity in the irrigation water of tested tomato plants. However, increasing the levels of leaf proline at higher salinity levels may improve salt stress tolerance by protecting the cell membrane, managing intercellular osmotic pressure, and preventing cell damage [20, 23]. This was attributed to the correlation analysis of the tested parameters with proline content.

As shown in Table 6, the proline content of leaves was highly (r = 0.835–0.870) correlated with Tr in grafted plants, whereas it was moderately (r = 0.633–0.720) correlated with Tr in non-grafted plants. Similar trends were observed for Pn and gs. However, the correlation between transpiration (Tr) proline content in the leaves was low (r = 0.478–0.551). Phosphorus and chlorophyll content of grafted and non-grafted plants. The behavior of the tested photosynthetic parameters indicated that in the presence of leaf proline, the chlorophyll cells were maintained well for the plants treated with saline water up to an EC of 6 dS m^-1^, whereas a decrease in the performance of the photosynthetic process was observed in plants irrigated with high saline water (9.5 dS m^-1^). Therefore, proline alleviates the destructive effects of salinity and effectively improves plant development.

**Table 6:** Correlation between proline and studied parameters.

	Grafted plants		Non-Grafted plants	
	DAT 60	DAT 120	DAT 60	DAT 120
**Salinity**	0.957	0.970	0.958	0.971
**Transpiration (Tr)**	-0.551	-0.478	-0.511	-0.549
**Stomatal conductance (gS)**	-0.782	-0.571	-0.639	-0.737
**Net photosynthesis rate (Pn)**	-0.738	-0.595	-0.825	-0.603
**Chlorophyll content of leaf**	-0.870	-0.835	-0.720	-0.633

## Conclusions

This study was conducted to investigate the influence of three salinity levels (2.5, 6.0, and 9.5 dS m^-1^) on leaf photosynthesis, chlorophyll content, and yield of three tomato cultivars (Valouro-RZ, Ghandora-F1, and Feisty-Red) grown in a hydroponic glasshouse. The conclusions are summarized as follows:

- The results indicated a significant (Pr<0.0001) negative effect of salinity on the studied leaf photosynthetic parameters, namely, transpiration rate (Tr), net photosynthetic rate (Pn), and stomatal conductance (gs). However, no significant differences were recorded in the leaf photosynthesis parameters of the studied tomato cultivars between the low (2.5 dS m^-1^) and medium (6.0 dS m^-1^) salinities. Grafting tomato plants onto the Maxifort rootstock induced a significant improvement in tomato plant leaf photosynthesis, with the accumulation of proline in tomato leaves.
- The chlorophyll content of the leaves was critically affected by salinity (Pr<0.0001) in all studied tomato cultivars. Where, the highest (48.99) and the lowest (43.95) mean values were recorded at the low (2.5 dS m^-1^) and high (9.5 dS m^-1^) salinities, respectively. In general, the cultivar Feisty-Red had the highest mean chlorophyll content value (47.96), followed by Valouro-RZ (46.28), while the lowest values were recorded for Ghandora-F1 (45.24). Moreover, the overall mean chlorophyll content for all varieties of grafted plants (46.84) was higher than that of the non-grafted plants (46.16).
- The results showed a negative effect of salinity on the total tomato fruit yield, with the highest (24.59 kg m^-2^) and lowest (17.92 kg m^-2^) mean fruit yields recorded at low and high salinities, respectively. However, the statistical results showed significant differences in tomato fruit yield only at high salinity compared with low and medium salinities for all tomato cultivars. No significant differences in tomato fruit yield were observed between low and medium salinities. Although grafting tomato plants onto the Maxifort rootstock improved the total fruit yield of the studied cultivars, no significant differences were observed in the total fruit yield between the non-grafted and grafted plants.
- Overall, all tomato cultivars studied could be successfully grown in a hydroponic system using saline water with a salt concentration of up to 6.0 dS m^-1^ without significantly affecting the total fruit yield.

## Supporting information

S1 Data Photosynthesis and yield data. (XLSX)

S2 Data Proline data. (XLSX)
